# Impact of marginalization on tobacco use in individuals diagnosed with head and neck Cancer

**DOI:** 10.1186/s40463-019-0380-5

**Published:** 2019-10-24

**Authors:** Grace Margaret Scott, Corliss Best, Kevin Fung, Michael Gupta, Doron D. Sommer, Christopher Szeto, Damian Christopher Micomonaco

**Affiliations:** 1 Department of Rural and Northern Health, Laurentian University, Sudbury, Canada; 2 Department of Otolaryngology-Head & Neck Surgery, University of Ottawa, Ottawa, Canada; 3 Department of Otolaryngology-Head & Neck Surgery, Western University, London, Canada; 4 Otolaryngology-Head & Neck Surgery Division, McMaster University-Dept. of Surgery, Hamilton, Canada; 5 Department of Otolaryngology-Head & Neck Surgery, Northern Ontario School of Medicine, Sudbury, Canada

**Keywords:** Smoking, Tobacco, Cancer, Addiction

## Abstract

**Background:**

Considerable evidence now indicates that individuals living in underprivileged neighbourhoods have higher rates of mortality and morbidity independent of individual-level characteristics. This study explored the impact of geographical marginalization on smoking cessation in a population of individuals with a diagnosis of head and neck cancer. The aims of this study were twofold: (1) assess the prevalence of smoking cessation in those with a previous diagnosis of head and neck cancer, (2) analyze the determinants of smoking alongside area-based measures of socioeconomic status.

**Methods:**

This was a cross-sectional study. We administered a self-reported nicotine dependence package to participants between the ages of 20–90 with a previous mucosal head and neck cancer diagnosis and with a history of tobacco use. Using the Canadian Marginalization (CAN-Marg) Index tool based on 2006 Canada Census data we compared the degree of marginalization to the smoking status. For those individuals who were currently smoking, nicotine dependence and readiness to quit were assessed. A summative score of marginalization was compared to smoking status of individuals.

**Results:**

The results from this study indicate that the summative level of marginalization developed from the combined factors of residential instability, material deprivation, ethnic concentration and dependency may be important factors in smoking cessation.

**Conclusions:**

This analysis of determinants of smoking alongside area-based measures of socioeconomic status may implicate the need for targeted population-based smoking cessation interventions.

## Introduction

There has been a recent interest in studying how contexts are related to the individual characteristics of geographic areas [1]. Area context has been shown to have significant relevance when it comes to forming policy. Furthermore, it becomes of particular importance when analyzing the determinants of health and mortality rates. It has been suggested that interventions to improve health should focus on efforts to improve neighbourhoods in socially disadvantaged areas [1, 2]. Individuals living in these areas have higher rates of mortality and morbidity independent of individual-level characteristics. This study explores the barriers to smoking cessation (SC) in individuals with a head and neck cancer diagnosis through an area-based perspective.

Smoking remains the number one cause of preventable death in Canada [3]. In cancer patients and survivors, the evidence is sufficient to infer a causal relationship between cigarette smoking and increased all-cause mortality and cancer-specific mortality [4]. Furthermore, in the field of head and neck cancer, the most important risk factors are tobacco and alcohol [5–10]. It is also well documented that SC after diagnosis improves prognostic outcomes [9]. Correspondingly, smoking cessation interventions in the perioperative period after a diagnosis of head and neck cancer have been shown to represent a unique opportunity for success [11]. Patients with head and neck cancer who continue to smoke after diagnosis and treatment are more likely to experience tumor recurrence and second primary malignancies. Previous research found that smoking during radiotherapy or chemoradiotherapy for head and neck cancer significantly reduced survival; however, patients who quit 12 weeks before treatment did not have significantly reduced survival [12]. A recent analysis from a randomized phase III trial of radiotherapy or chemoradiotherapy found that current smoking during treatment significantly decreased disease-specific and overall survival in patients with head and neck cancer, and that smoking substantially worsened the otherwise favorable prognosis of human papillomavirus-driven head and neck cancer [13, 14]. Beyond radiotherapy and chemotherapy, smokers compared with nonsmokers and former smokers compared with those who never smoked have more postoperative healing complications. Additionally, perioperative smoking cessation reduces surgical site infections [15]. Although the diagnosis of a tobacco related malignancy clearly represents a strong catalyst for SC, a sizable subgroup of patients continue to smoke [16].

Due to the importance of cessation, and the various barriers to success in this endeavour, we aimed to study some of these aspects with respect to socioeconomic variation. The main aim of this study was to analyze the determinants of smoking alongside area-based measures of socioeconomic status.

## Methods

This was a cross-sectional study and included consecutive English speaking head and neck cancer patients between the ages of 20–90. Participants were recruited from four different sites in Ontario between 2013 and 2017. This included two northern Ontario sites (Sudbury and Sault Ste. Marie) and two southern Ontario sites (London and Hamilton). We included all of those with a mucosal head and neck cancer diagnosis (excluding melanoma) with a history of tobacco use. Tobacco use was identified by a retrospective review of clinic charts/notes.

### Measurement instruments

We administered a demographic questionnaire and for those participants who were currently smoking we administered the CAMH readiness ruler [17] and the Fagerstom test [18] for nicotine dependence. All questionnaires were completed over the telephone.

### CAN-Marg

In order to address our second objective and analyze the determinants of smoking alongside area-based measures of socioeconomic status we used the Canadian Marginalization Index [19]. CAN-Marg is a census-based, empirically derived and theoretically informed tool designed to reflect a broader conceptualization of Canadian marginalization. The index has demonstrated marked stability across time and geographic area. Each of the four dimensions show strong and significant associations with selected health and behavioural issues, and these associations differ depending on which of the dimensions of marginalization is examined. The four dimensions of the tool are residential instability, material deprivation, ethnic concentration and dependency. Each group contains a fifth of the geographic units. A summative marginalization score can be calculated as the mean quintile value of each of these 4 subdimensions.

### Statistical analysis

Alongside descriptive statistics, a Mann-Whitney test was conducted to assess differences between groups. Significance was defined at *p* < 0.05. All data were analyzed using SPSS 22.0 for Windows (IBM Corp., 2013).

## Results

In total, 278 individuals were invited to participate in this study. Complete questionnaires were received from 113 participates (40.6% response rate). This included 28 (24.8%) current smokers and 85 (71.2%) previous smokers. The total number of participants recruited from each site was as follows: Sault Ste. Marie (*n* = 34 (30.1%)), Sudbury (*n* = 50 (44.2%)), London (*n* = 28 (24.8%)), and Hamilton (n = 5 (4.4%)). A complete breakdown of demographics by smoking status can be found in Table 1. Of note, the 3 participants with ‘other’ cancer sites included two pyriform sinus (hypopharyx) and a nasal septal lesion.

**Table 1:** Demographic Information

Variable		Total n (%)	
		Current Smokers	Reformed Smokers
Number of Participants		28 (24.8)	85 (75.2)
Men		18 (64.3)	66 (77.6)
Women		10 (35.7)	19 (22.4)
Age	Mean –	67.95	63.29
Cancer Site			
Oral Cavity		15 (53.6)	32 (37.6)
Larynx		5 (17.9)	33 (38.8)
Oropharynx		7 (25)	18 (21.2)
Other		1 (3.6)	2 (2.4)
Treatment Type			
Surgery		15 (53.6)	62 (72.9)
Radiation		21 (75)	55 (64.7)
Chemotherapy		14 (50)	25 (29.4)
Marital Status			
Married		13 (46.4)	52 (61.2)
Divorced		1 (3.6)	11 (12.9)
Separated		0 (0)	1 (1.2)
Widowed		6 (21.4)	4 (4.7)
Common-Law		1 (3.6)	10 (11.8)
Single		5 (17.9)	6 (7.1)
Undisclosed		2 (7.1)	1 (1.2)
Occupational Status			
Full Time		3 (10.7)	15 (17.6)
Part Time		1 (3.6)	2 (2.4)
Retired		12 (42.9)	57 (67)
Disability		10 (35.7)	8 (9.4)
Undisclosed		2 (7.1)	3 (3.5)

**Fig. 1:**
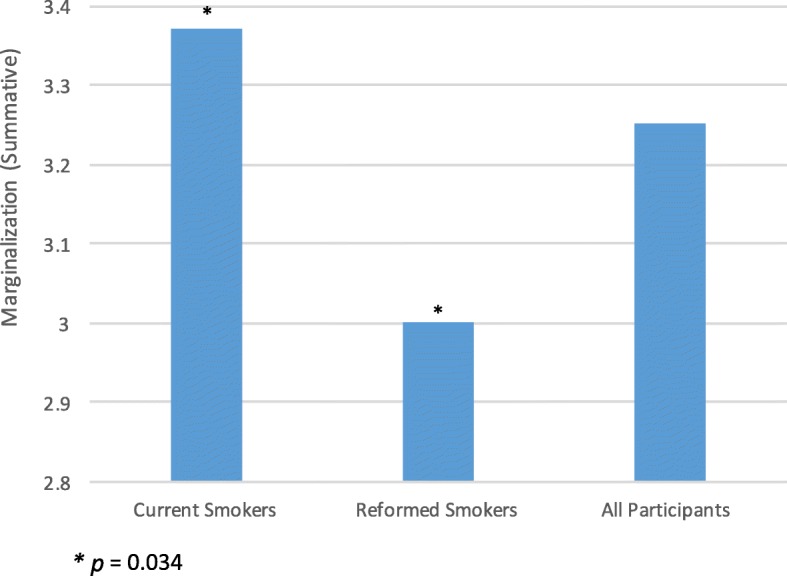
Summative marginalization scores across Ontario

A Mann-Whitney U Test was conducted for summative marginalization scores and compared current smokers to reformed smokers. This test revealed a significant difference in summative marginalization scores of current smokers and reformed smokers as shown in Fig. 1.

Using Cohen’s measure of effect sizes, we see a small effect in both of these cases as denoted by an r-value of .19 for summative marginalization.

The results from the participants that continued to smoke yielded a mean score of 4.3 (out of 10) on the Fagerstom test for nicotine dependence. A higher score on this test indicates a great level of dependence. A score of 4.3 indicates a moderate level of dependence. The readiness ruler gauged the level of importance, confidence and readiness to quit smoking as a score out of 10. On this test, the smokers had mean importance, confidence and readiness scores of 6.3, 5.1, and 4.9 respectively.

**Fig. 2:**
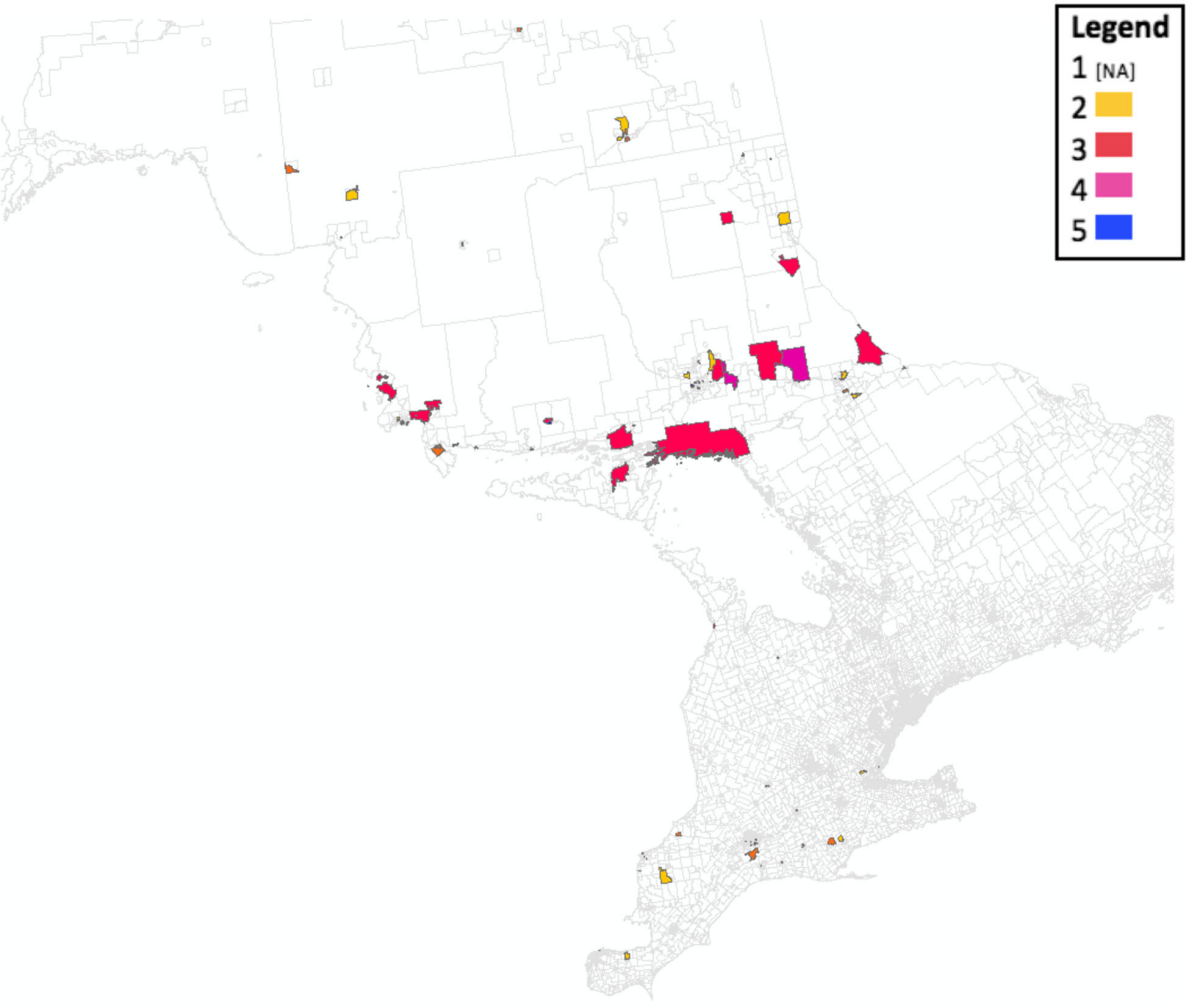
Summative marginalization across Ontario

Using geographic information system mapping, we were able to plot the summative marginalization scores from all of the dissemination areas of the participants. A higher summative marginalization score indicates a greater level of marginalization in that area. The scale ranges from 1 to 5, with 1 being the lowest level of marginalization and 5 the highest. Figure 2 depicts a visual representation of respondents by geographic area. The areas on the map that remain blank represent those dissemination areas not represented by participants in this study. Additionally, no participants in this study received a summative score of 1 (indicating the lowest level of marginalization).

## Discussion

If we are to construct effective policy responses to health inequalities, especially at the local level, then it is essential that these be informed by an understanding of the potential importance of the geographic factors [2]. Amongst other factors, these contextual effects would appear to impact patients’ intention to quit smoking, as indicated by our results. Participants that continue to smoke tended to live in more marginalized communities as compared to those that have successfully quit smoking.

The present study found that 24.8% (*n* = 28) of participants continued to smoke after a diagnosis of head and neck cancer. A similar study examined lifestyle behaviours in a heterogeneous group of adult cancer survivors and found that since cancer diagnosis, 46% of smokers quit smoking and 47% improved their dietary habits [20]. They also found that these adult cancer survivors who changed their lifestyle behaviors varied greatly and specifically related this to demographic variables [20]. The higher quit rate in our study may highlight the unique factors associated with a head and neck cancer diagnosis as compared to other cancer types. Though other cancer sites are associated with tobacco abuse, smoking unequivocally causes head and neck cancer [21]. The implicit tie between smoking and head and neck cancer indicates the need for targeted smoking cessation interventions [22]. Although a majority of health care providers acknowledge that smoking cessation is within their scope of practice, many are unsure of their specific role [23].

There are several limitations to acknowledge in this study. As a cross-sectional study, the results are reflective of one distinctive point in time and this may limit the generalizability of the study findings. All participants included in this study had a history of tobacco use. Because of this, there is no way of knowing whether non-smokers would have displayed a similar pattern of marginalization. We are also unable to extract baseline smoking rates for these geographical regions and are unable to compare the rate of smoking in the study population to baseline rates by geographic area. Additionally, geographic area based on residential postal code was used as a surrogate marker for marginalization in this population. As an area-based measure of geographic marginalization, the marginalization scores identified for each participant may not reflect the actual level of individual marginalization. Futhermore, this was a voluntary, self-reporting study. In this case, the results are subject to both selection and response bias. Individuals may have been excluded from being invited to participate in this study based on their response to tobacco use questioning.

## Conclusion

Though SC may be important to individuals who continue to smoke after a cancer diagnosis, they may not feel ready or confident in their ability to quit. Summative level of marginalization developed from the combined factors of residential instability, material deprivation, ethnic influences and dependency may be a critical factor in SC. Our goal was to further develop an understanding of the moderating factors behind one’s decision to continue to smoke after a diagnosis of head and neck cancer and whether the social determinants of health such as neighbourhood socioeconomic deprivation play a role. Despite having the same access to regional cancer centres, oncologists, SC education and a multidisciplinary cancer team as per Cancer Care Ontario’s (provincial governmental advisor on cancer care) rules and regulations, there remain intrinsic differences in smoking behaviours based on area based socio-economic status. This may suggest a need for specific targeted population-based SC interventions.

## Data Availability

The datasets generated and/or analysed during the current study are not publicly available due to confidentialty, but are available from the corresponding author on reasonable request.
